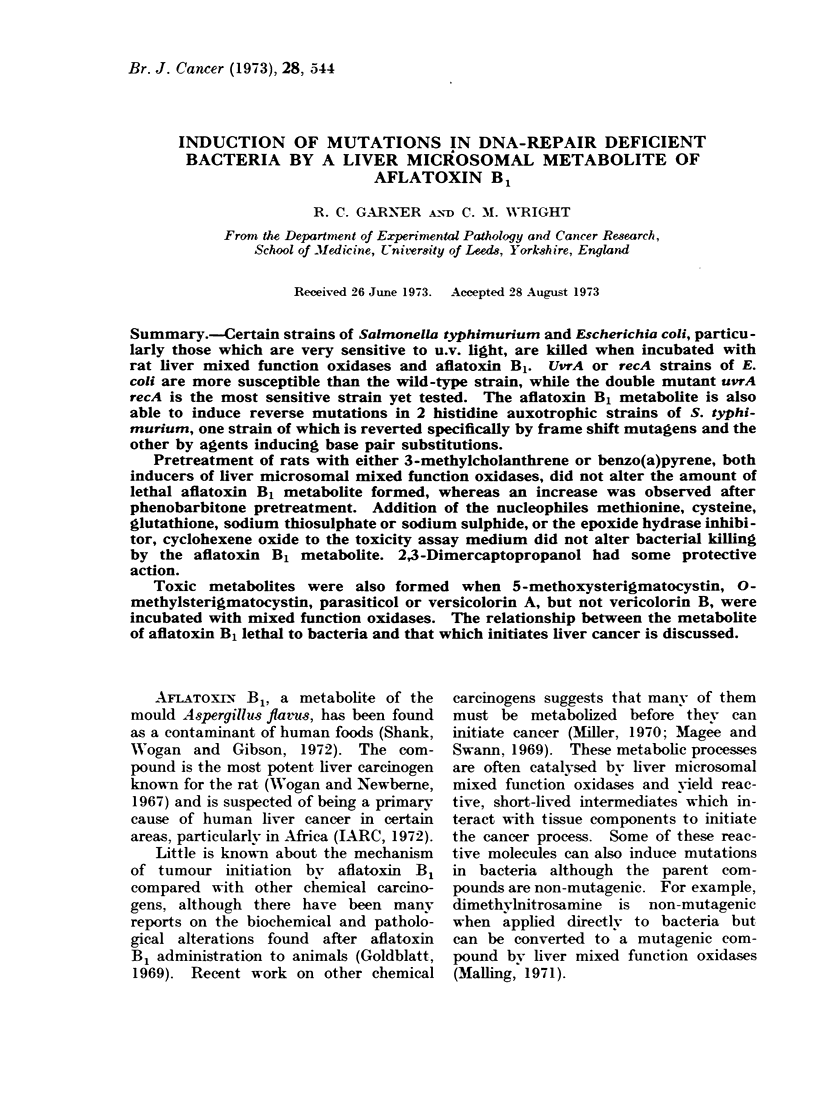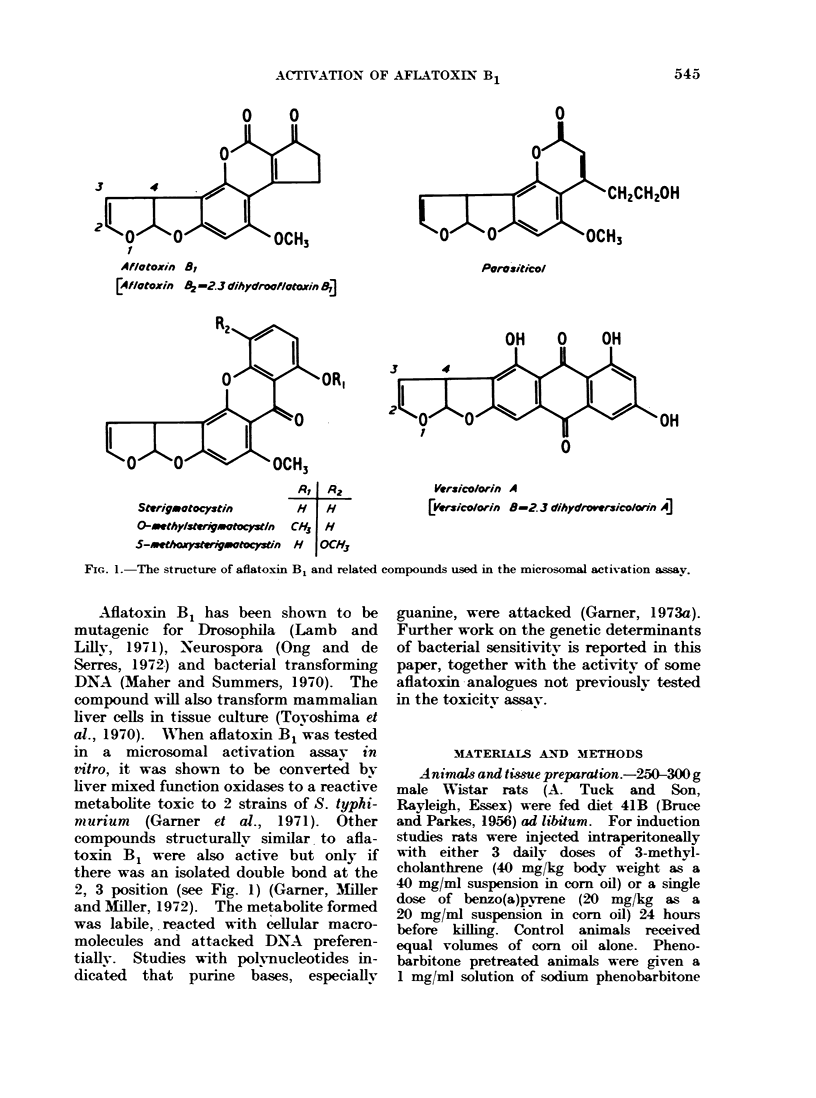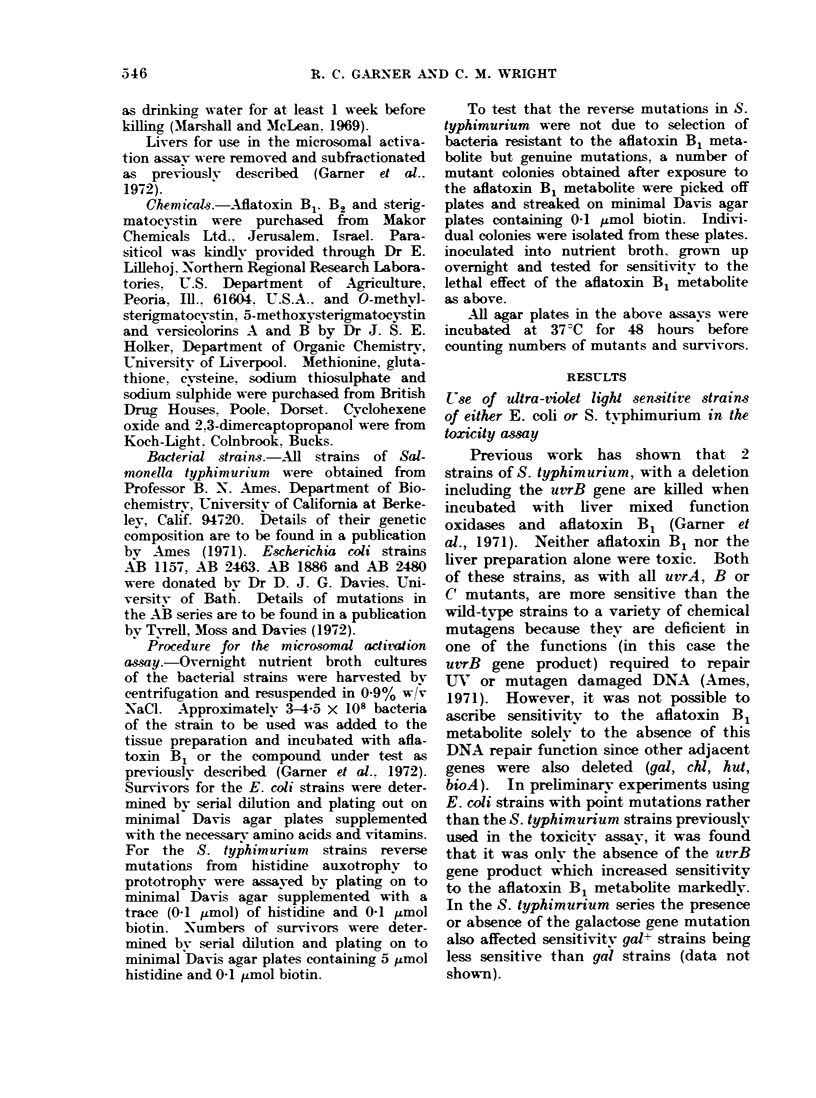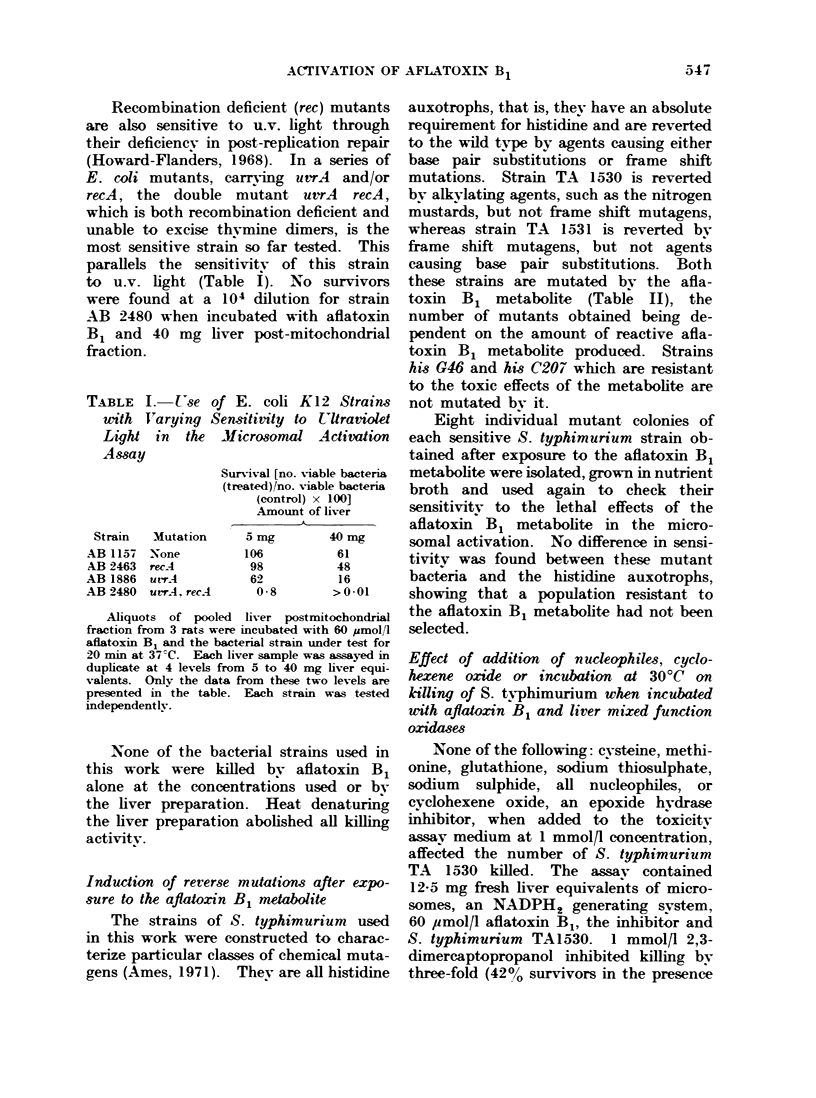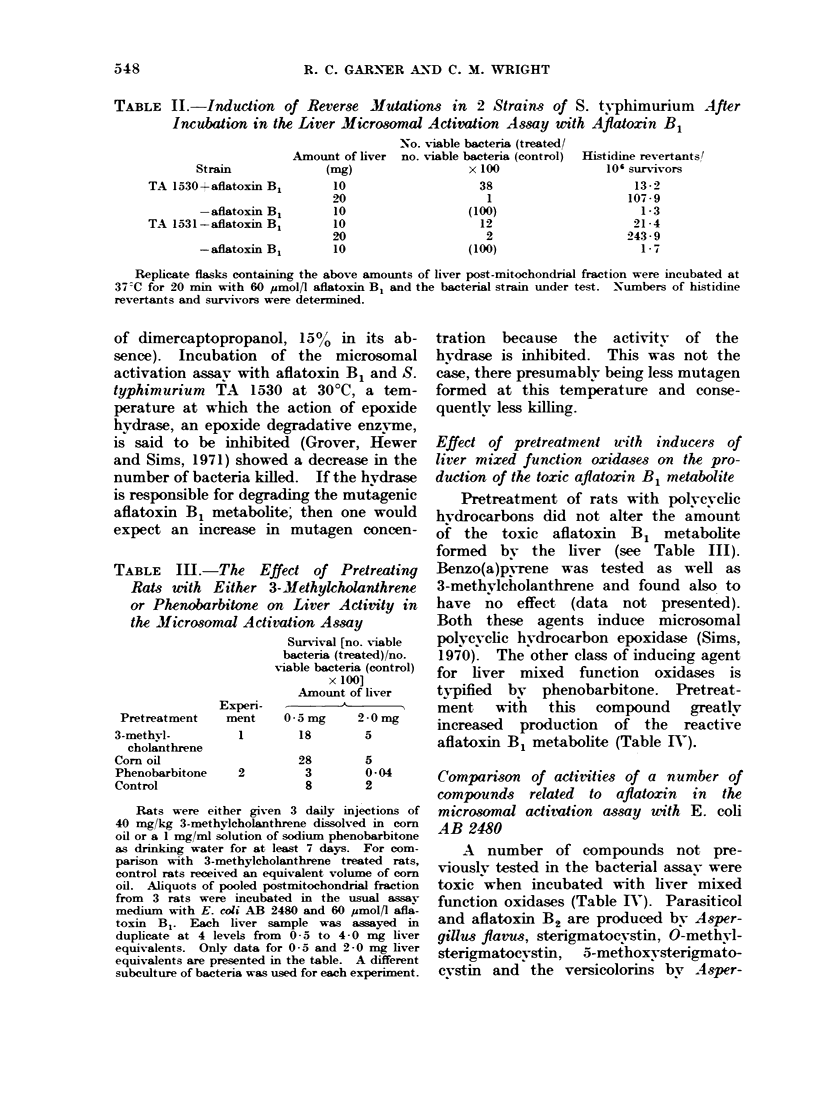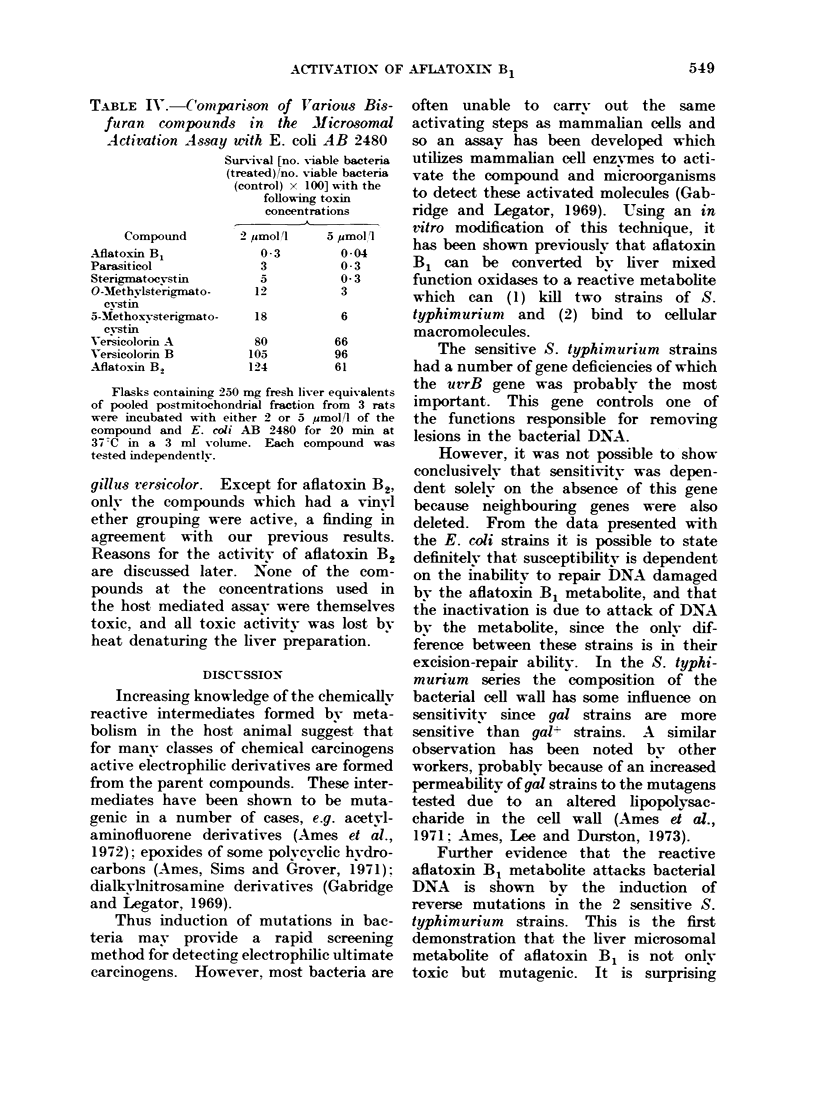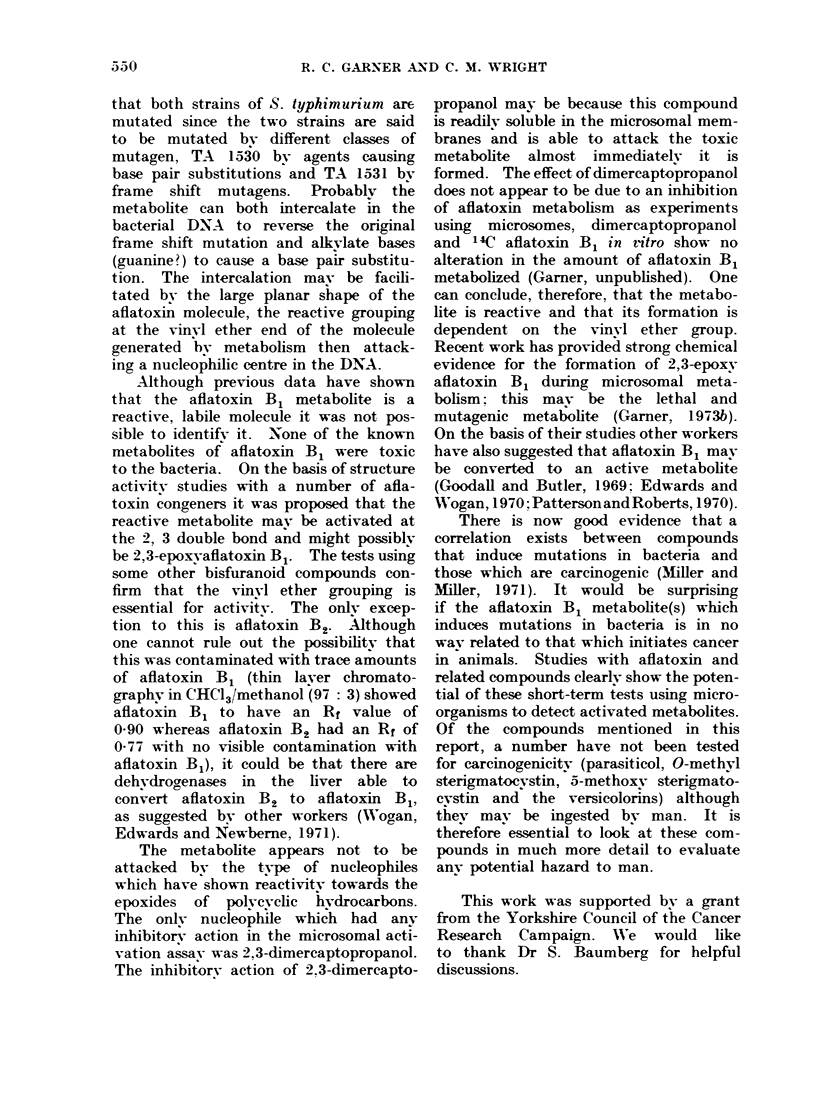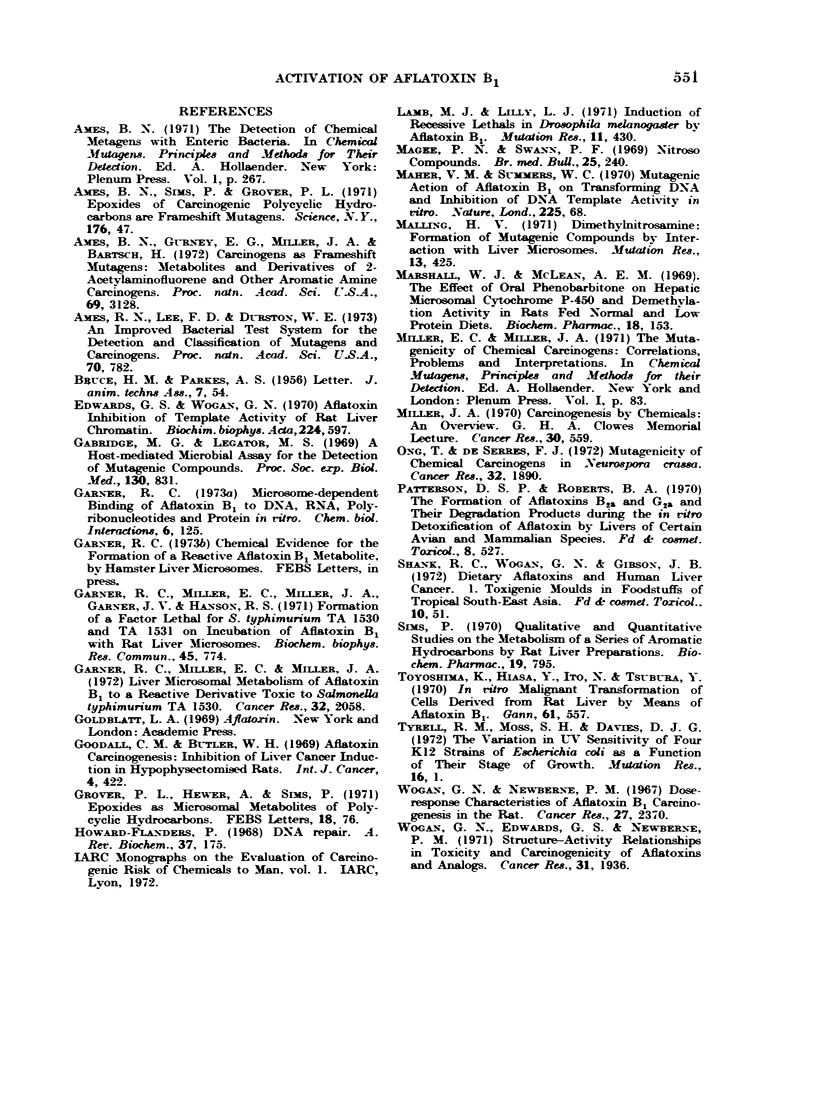# Induction of Mutations in DNA-repair Deficient Bacteria by a Liver Microsomal Metabolite of Aflatoxin B_1_

**DOI:** 10.1038/bjc.1973.184

**Published:** 1973-12

**Authors:** R. C. Garner, C. M. Wright

## Abstract

Certain strains of *Salmonella typhimurium* and *Escherichia coli,* particularly those which are very sensitive to u.v. light, are killed when incubated with rat liver mixed function oxidases and aflatoxin B_1_. *UvrA* or *recA* strains of *E. coli* are more susceptible than the wild-type strain, while the double mutant *uvrA recA* is the most sensitive strain yet tested. The aflatoxin B_1_ metabolite is also able to induce reverse mutations in 2 histidine auxotrophic strains of *S. typhimurium,* one strain of which is reverted specifically by frame shift mutagens and the other by agents inducing base pair substitutions.

Pretreatment of rats with either 3-methylcholanthrene or benzo(a)pyrene, both inducers of liver microsomal mixed function oxidases, did not alter the amount of lethal aflatoxin B_1_ metabolite formed, whereas an increase was observed after phenobarbitone pretreatment. Addition of the nucleophiles methionine, cysteine, glutathione, sodium thiosulphate or sodium sulphide, or the epoxide hydrase inhibitor, cyclohexene oxide to the toxicity assay medium did not alter bacterial killing by the aflatoxin B_1_ metabolite. 2,3-Dimercaptopropanol had some protective action.

Toxic metabolites were also formed when 5-methoxysterigmatocystin, *O*-methylsterigmatocystin, parasiticol or versicolorin A, but not vericolorin B, were incubated with mixed function oxidases. The relationship between the metabolite of aflatoxin B_1_ lethal to bacteria and that which initiates liver cancer is discussed.


Br. J. Cancer (1973), 28, 544

### INDUCTION OF MUTATIONS IN DNA-REPAIR DEFICIENT BACTERIA BY A LIVER MICROSOMAL METABOLITE OF AFLATOXIN $B_1$

R. C. GARNER AND C. M. WRIGHT

*From the Department of Experimental Pathology and Cancer Research, School of Medicine, University of Leeds, Yorkshire, England*

Received 26 June 1973. Accepted 28 August 1973

**Summary.—Certain strains of *Salmonella typhimurium* and *Escherichia coli*, particularly those which are very sensitive to u.v. light, are killed when incubated with rat liver mixed function oxidases and aflatoxin $B_1$. *UvrA* or *recA* strains of *E. coli* are more susceptible than the wild-type strain, while the double mutant *uvrA recA* is the most sensitive strain yet tested. The aflatoxin $B_1$ metabolite is also able to induce reverse mutations in 2 histidine auxotrophic strains of *S. typhimurium*, one strain of which is reverted specifically by frame shift mutagens and the other by agents inducing base pair substitutions.**

**Pretreatment of rats with either 3-methylcholanthrene or benzo(a)pyrene, both inducers of liver microsomal mixed function oxidases, did not alter the amount of lethal aflatoxin $B_1$ metabolite formed, whereas an increase was observed after phenobarbitone pretreatment. Addition of the nucleophiles methionine, cysteine, glutathione, sodium thiosulphate or sodium sulphide, or the epoxide hydrase inhibitor, cyclohexene oxide to the toxicity assay medium did not alter bacterial killing by the aflatoxin $B_1$ metabolite. 2,3-Dimercaptopropanol had some protective action.**

**Toxic metabolites were also formed when 5-methoxysterigmatocystin, *O*-methylsterigmatocystin, parasiticol or versicolorin A, but not vericolorin B, were incubated with mixed function oxidases. The relationship between the metabolite of aflatoxin $B_1$ lethal to bacteria and that which initiates liver cancer is discussed.**

AFLATOXIN $B_1$, a metabolite of the mould *Aspergillus flavus*, has been found as a contaminant of human foods (Shank, Wogan and Gibson, 1972). The compound is the most potent liver carcinogen known for the rat (Wogan and Newberne, 1967) and is suspected of being a primary cause of human liver cancer in certain areas, particularly in Africa (IARC, 1972).

Little is known about the mechanism of tumour initiation by aflatoxin $B_1$ compared with other chemical carcinogens, although there have been many reports on the biochemical and pathological alterations found after aflatoxin $B_1$ administration to animals (Goldblatt, 1969). Recent work on other chemical carcinogens suggests that many of them must be metabolized before they can initiate cancer (Miller, 1970; Magee and Swann, 1969). These metabolic processes are often catalysed by liver microsomal mixed function oxidases and yield reactive, short-lived intermediates which interact with tissue components to initiate the cancer process. Some of these reactive molecules can also induce mutations in bacteria although the parent compounds are non-mutagenic. For example, dimethylnitrosamine is non-mutagenic when applied directly to bacteria but can be converted to a mutagenic compound by liver mixed function oxidases (Malling, 1971).

FIG. 1.—The structure of aflatoxin $B_1$ and related compounds used in the microsomal activation assay.

Aflatoxin $B_1$ has been shown to be mutagenic for Drosophila (Lamb and Lilly, 1971), Neurospora (Ong and de Serres, 1972) and bacterial transforming DNA (Maher and Summers, 1970). The compound will also transform mammalian liver cells in tissue culture (Toyoshima *et al.*, 1970). When aflatoxin $B_1$ was tested in a microsomal activation assay *in vitro*, it was shown to be converted by liver mixed function oxidases to a reactive metabolite toxic to 2 strains of *S. typhimurium* (Garner *et al.*, 1971). Other compounds structurally similar to aflatoxin $B_1$ were also active but only if there was an isolated double bond at the 2, 3 position (see Fig. 1) (Garner, Miller and Miller, 1972). The metabolite formed was labile, reacted with cellular macromolecules and attacked DNA preferentially. Studies with polynucleotides indicated that purine bases, especially guanine, were attacked (Garner, 1973*a*). Further work on the genetic determinants of bacterial sensitivity is reported in this paper, together with the activity of some aflatoxin analogues not previously tested in the toxicity assay.

### MATERIALS AND METHODS

*Animals and tissue preparation.*—250–300 g male Wistar rats (A. Tuck and Son, Rayleigh, Essex) were fed diet 41B (Bruce and Parkes, 1956) *ad libitum*. For induction studies rats were injected intraperitoneally with either 3 daily doses of 3-methylcholanthrene (40 mg/kg body weight as a 40 mg/ml suspension in corn oil) or a single dose of benzo(a)pyrene (20 mg/kg as a 20 mg/ml suspension in corn oil) 24 hours before killing. Control animals received equal volumes of corn oil alone. Phenobarbitone pretreated animals were given a 1 mg/ml solution of sodium phenobarbitone as drinking water for at least 1 week before killing (Marshall and McLean, 1969).

Livers for use in the microsomal activation assay were removed and subfractionated as previously described (Garner *et al.*, 1972).

*Chemicals.*—Aflatoxin $B_1$, $B_2$ and sterigmatocystin were purchased from Makor Chemicals Ltd., Jerusalem, Israel. Parasiticol was kindly provided through Dr E. Lillehoj, Northern Regional Research Laboratories, U.S. Department of Agriculture, Peoria, Ill., 61604, U.S.A., and *O*-methylsterigmatocystin, 5-methoxysterigmatocystin and versicolorins A and B by Dr J. S. E. Holker, Department of Organic Chemistry, University of Liverpool. Methionine, glutathione, cysteine, sodium thiosulphate and sodium sulphide were purchased from British Drug Houses, Poole, Dorset. Cyclohexene oxide and 2,3-dimercaptopropanol were from Koch-Light, Colnbrook, Bucks.

*Bacterial strains.*—All strains of *Salmonella typhimurium* were obtained from Professor B. N. Ames, Department of Biochemistry, University of California at Berkeley, Calif. 94720. Details of their genetic composition are to be found in a publication by Ames (1971). *Escherichia coli* strains AB 1157, AB 2463, AB 1886 and AB 2480 were donated by Dr D. J. G. Davies, University of Bath. Details of mutations in the AB series are to be found in a publication by Tyrell, Moss and Davies (1972).

*Procedure for the microsomal activation assay.*—Overnight nutrient broth cultures of the bacterial strains were harvested by centrifugation and resuspended in 0·9% w/v NaCl. Approximately $3–4·5 \times 10^8$ bacteria of the strain to be used was added to the tissue preparation and incubated with aflatoxin $B_1$ or the compound under test as previously described (Garner *et al.*, 1972). Survivors for the *E. coli* strains were determined by serial dilution and plating out on minimal Davis agar plates supplemented with the necessary amino acids and vitamins. For the *S. typhimurium* strains reverse mutations from histidine auxotrophy to prototrophy were assayed by plating on to minimal Davis agar supplemented with a trace (0·1 $\mu$mol) of histidine and 0·1 $\mu$mol biotin. Numbers of survivors were determined by serial dilution and plating on to minimal Davis agar plates containing 5 $\mu$mol histidine and 0·1 $\mu$mol biotin.

To test that the reverse mutations in *S. typhimurium* were not due to selection of bacteria resistant to the aflatoxin $B_1$ metabolite but genuine mutations, a number of mutant colonies obtained after exposure to the aflatoxin $B_1$ metabolite were picked off plates and streaked on minimal Davis agar plates containing 0·1 $\mu$mol biotin. Individual colonies were isolated from these plates, inoculated into nutrient broth, grown up overnight and tested for sensitivity to the lethal effect of the aflatoxin $B_1$ metabolite as above.

All agar plates in the above assays were incubated at 37°C for 48 hours before counting numbers of mutants and survivors.

### RESULTS

### *Use of ultra-violet light sensitive strains of either* E. coli *or* S. typhimurium *in the toxicity assay*

Previous work has shown that 2 strains of *S. typhimurium*, with a deletion including the *uvrB* gene are killed when incubated with liver mixed function oxidases and aflatoxin $B_1$ (Garner *et al.*, 1971). Neither aflatoxin $B_1$ nor the liver preparation alone were toxic. Both of these strains, as with all *uvrA*, *B* or *C* mutants, are more sensitive than the wild-type strains to a variety of chemical mutagens because they are deficient in one of the functions (in this case the *uvrB* gene product) required to repair UV or mutagen damaged DNA (Ames, 1971). However, it was not possible to ascribe sensitivity to the aflatoxin $B_1$ metabolite solely to the absence of this DNA repair function since other adjacent genes were also deleted (*gal*, *chl*, *hut*, *bioA*). In preliminary experiments using *E. coli* strains with point mutations rather than the *S. typhimurium* strains previously used in the toxicity assay, it was found that it was only the absence of the *uvrB* gene product which increased sensitivity to the aflatoxin $B_1$ metabolite markedly. In the *S. typhimurium* series the presence or absence of the galactose gene mutation also affected sensitivity $gal^+$ strains being less sensitive than *gal* strains (data not shown).

Recombination deficient (*rec*) mutants are also sensitive to u.v. light through their deficiency in post-replication repair (Howard-Flanders, 1968). In a series of *E. coli* mutants, carrying *uvrA* and/or *recA*, the double mutant *uvrA recA*, which is both recombination deficient and unable to excise thymine dimers, is the most sensitive strain so far tested. This parallels the sensitivity of this strain to u.v. light (Table I). No survivors were found at a $10^4$ dilution for strain AB 2480 when incubated with aflatoxin $B_1$ and 40 mg liver post-mitochondrial fraction.

TABLE I.—*Use of* E. coli *K12 Strains with Varying Sensitivity to Ultraviolet Light in the Microsomal Activation Assay*

| | | Survival [no. viable bacteria (treated)/no. viable bacteria (control) × 100] Amount of liver | |
|---|---|---|---|
| Strain | Mutation | 5 mg | 40 mg |
| AB 1157 | None | 106 | 61 |
| AB 2463 | *recA* | 98 | 48 |
| AB 1886 | *uvrA* | 62 | 16 |
| AB 2480 | *uvrA, recA* | 0·8 | >0·01 |

Aliquots of pooled liver postmitochondrial fraction from 3 rats were incubated with 60 μmol/l aflatoxin $B_1$ and the bacterial strain under test for 20 min at 37°C. Each liver sample was assayed in duplicate at 4 levels from 5 to 40 mg liver equivalents. Only the data from these two levels are presented in the table. Each strain was tested independently.

None of the bacterial strains used in this work were killed by aflatoxin $B_1$ alone at the concentrations used or by the liver preparation. Heat denaturing the liver preparation abolished all killing activity.

*Induction of reverse mutations after exposure to the aflatoxin $B_1$ metabolite*

The strains of *S. typhimurium* used in this work were constructed to characterize particular classes of chemical mutagens (Ames, 1971). They are all histidine auxotrophs, that is, they have an absolute requirement for histidine and are reverted to the wild type by agents causing either base pair substitutions or frame shift mutations. Strain TA 1530 is reverted by alkylating agents, such as the nitrogen mustards, but not frame shift mutagens, whereas strain TA 1531 is reverted by frame shift mutagens, but not agents causing base pair substitutions. Both these strains are mutated by the aflatoxin $B_1$ metabolite (Table II), the number of mutants obtained being dependent on the amount of reactive aflatoxin $B_1$ metabolite produced. Strains *his G46* and *his C207* which are resistant to the toxic effects of the metabolite are not mutated by it.

Eight individual mutant colonies of each sensitive *S. typhimurium* strain obtained after exposure to the aflatoxin $B_1$ metabolite were isolated, grown in nutrient broth and used again to check their sensitivity to the lethal effects of the aflatoxin $B_1$ metabolite in the microsomal activation. No difference in sensitivity was found between these mutant bacteria and the histidine auxotrophs, showing that a population resistant to the aflatoxin $B_1$ metabolite had not been selected.

*Effect of addition of nucleophiles, cyclohexene oxide or incubation at 30°C on killing of* S. typhimurium *when incubated with aflatoxin $B_1$ and liver mixed function oxidases*

None of the following: cysteine, methionine, glutathione, sodium thiosulphate, sodium sulphide, all nucleophiles, or cyclohexene oxide, an epoxide hydrase inhibitor, when added to the toxicity assay medium at 1 mmol/l concentration, affected the number of *S. typhimurium* TA 1530 killed. The assay contained 12·5 mg fresh liver equivalents of microsomes, an $NADPH_2$ generating system, 60 μmol/l aflatoxin $B_1$, the inhibitor and *S. typhimurium* TA1530. 1 mmol/l 2,3-dimercaptopropanol inhibited killing by three-fold (42% survivors in the presence

TABLE II.—*Induction of Reverse Mutations in 2 Strains of* S. typhimurium *After Incubation in the Liver Microsomal Activation Assay with Aflatoxin $B_1$*

| Strain | Amount of liver (mg) | No. viable bacteria (treated/ no. viable bacteria (control) ×100 | Histidine revertants/ $10^6$ survivors |
|---|---|---|---|
| TA 1530 + aflatoxin $B_1$ | 10 | 38 | 13·2 |
| | 20 | 1 | 107·9 |
| − aflatoxin $B_1$ | 10 | (100) | 1·3 |
| TA 1531 + aflatoxin $B_1$ | 10 | 12 | 21·4 |
| | 20 | 2 | 243·9 |
| − aflatoxin $B_1$ | 10 | (100) | 1·7 |

Replicate flasks containing the above amounts of liver post-mitochondrial fraction were incubated at 37°C for 20 min with 60 μmol/l aflatoxin $B_1$ and the bacterial strain under test. Numbers of histidine revertants and survivors were determined.

of dimercaptopropanol, 15% in its absence). Incubation of the microsomal activation assay with aflatoxin $B_1$ and *S. typhimurium* TA 1530 at 30°C, a temperature at which the action of epoxide hydrase, an epoxide degradative enzyme, is said to be inhibited (Grover, Hewer and Sims, 1971) showed a decrease in the number of bacteria killed. If the hydrase is responsible for degrading the mutagenic aflatoxin $B_1$ metabolite, then one would expect an increase in mutagen concentration because the activity of the hydrase is inhibited. This was not the case, there presumably being less mutagen formed at this temperature and consequently less killing.

TABLE III.—*The Effect of Pretreating Rats with Either 3-Methylcholanthrene or Phenobarbitone on Liver Activity in the Microsomal Activation Assay*

| Pretreatment | Experiment | Survival [no. viable bacteria (treated)/no. viable bacteria (control) ×100] Amount of liver 0·5 mg | 2·0 mg |
|---|---|---|---|
| 3-methyl-cholanthrene | 1 | 18 | 5 |
| Corn oil | | 28 | 5 |
| Phenobarbitone | 2 | 3 | 0·04 |
| Control | | 8 | 2 |

Rats were either given 3 daily injections of 40 mg/kg 3-methylcholanthrene dissolved in corn oil or a 1 mg/ml solution of sodium phenobarbitone as drinking water for at least 7 days. For comparison with 3-methylcholanthrene treated rats, control rats received an equivalent volume of corn oil. Aliquots of pooled postmitochondrial fraction from 3 rats were incubated in the usual assay medium with *E. coli* AB 2480 and 60 μmol/l aflatoxin $B_1$. Each liver sample was assayed in duplicate at 4 levels from 0·5 to 4·0 mg liver equivalents. Only data for 0·5 and 2·0 mg liver equivalents are presented in the table. A different subculture of bacteria was used for each experiment.

*Effect of pretreatment with inducers of liver mixed function oxidases on the production of the toxic aflatoxin $B_1$ metabolite*

Pretreatment of rats with polycyclic hydrocarbons did not alter the amount of the toxic aflatoxin $B_1$ metabolite formed by the liver (see Table III). Benzo(a)pyrene was tested as well as 3-methylcholanthrene and found also to have no effect (data not presented). Both these agents induce microsomal polycyclic hydrocarbon epoxidase (Sims, 1970). The other class of inducing agent for liver mixed function oxidases is typified by phenobarbitone. Pretreatment with this compound greatly increased production of the reactive aflatoxin $B_1$ metabolite (Table IV).

*Comparison of activities of a number of compounds related to aflatoxin in the microsomal activation assay with* E. coli *AB 2480*

A number of compounds not previously tested in the bacterial assay were toxic when incubated with liver mixed function oxidases (Table IV). Parasiticol and aflatoxin $B_2$ are produced by *Aspergillus flavus*, sterigmatocystin, *O*-methylsterigmatocystin, 5-methoxysterigmatocystin and the versicolorins by *Asper-*

TABLE IV.—*Comparison of Various Bisfuran compounds in the Microsomal Activation Assay with* E. coli *AB* 2480

| Compound | Survival [no. viable bacteria (treated)/no. viable bacteria (control) × 100] with the following toxin concentrations | |
|---|---|---|
| | 2 μmol/l | 5 μmol/l |
| Aflatoxin $B_1$ | 0·3 | 0·04 |
| Parasiticol | 3 | 0·3 |
| Sterigmatocystin | 5 | 0·3 |
| *O*-Methylsterigmatocystin | 12 | 3 |
| 5-Methoxysterigmatocystin | 18 | 6 |
| Versicolorin A | 80 | 66 |
| Versicolorin B | 105 | 96 |
| Aflatoxin $B_2$ | 124 | 61 |

Flasks containing 250 mg fresh liver equivalents of pooled postmitochondrial fraction from 3 rats were incubated with either 2 or 5 μmol/l of the compound and *E. coli* AB 2480 for 20 min at 37°C in a 3 ml volume. Each compound was tested independently.

*gillus versicolor*. Except for aflatoxin $B_2$, only the compounds which had a vinyl ether grouping were active, a finding in agreement with our previous results. Reasons for the activity of aflatoxin $B_2$ are discussed later. None of the compounds at the concentrations used in the host mediated assay were themselves toxic, and all toxic activity was lost by heat denaturing the liver preparation.

### DISCUSSION

Increasing knowledge of the chemically reactive intermediates formed by metabolism in the host animal suggest that for many classes of chemical carcinogens active electrophilic derivatives are formed from the parent compounds. These intermediates have been shown to be mutagenic in a number of cases, *e.g.* acetylaminofluorene derivatives (Ames *et al.*, 1972); epoxides of some polycyclic hydrocarbons (Ames, Sims and Grover, 1971); dialkylnitrosamine derivatives (Gabridge and Legator, 1969).

Thus induction of mutations in bacteria may provide a rapid screening method for detecting electrophilic ultimate carcinogens. However, most bacteria are often unable to carry out the same activating steps as mammalian cells and so an assay has been developed which utilizes mammalian cell enzymes to activate the compound and microorganisms to detect these activated molecules (Gabridge and Legator, 1969). Using an *in vitro* modification of this technique, it has been shown previously that aflatoxin $B_1$ can be converted by liver mixed function oxidases to a reactive metabolite which can (1) kill two strains of *S. typhimurium* and (2) bind to cellular macromolecules.

The sensitive *S. typhimurium* strains had a number of gene deficiencies of which the *uvrB* gene was probably the most important. This gene controls one of the functions responsible for removing lesions in the bacterial DNA.

However, it was not possible to show conclusively that sensitivity was dependent solely on the absence of this gene because neighbouring genes were also deleted. From the data presented with the *E. coli* strains it is possible to state definitely that susceptibility is dependent on the inability to repair DNA damaged by the aflatoxin $B_1$ metabolite, and that the inactivation is due to attack of DNA by the metabolite, since the only difference between these strains is in their excision-repair ability. In the *S. typhimurium* series the composition of the bacterial cell wall has some influence on sensitivity since *gal* strains are more sensitive than *gal*$^+$ strains. A similar observation has been noted by other workers, probably because of an increased permeability of *gal* strains to the mutagens tested due to an altered lipopolysaccharide in the cell wall (Ames *et al.*, 1971; Ames, Lee and Durston, 1973).

Further evidence that the reactive aflatoxin $B_1$ metabolite attacks bacterial DNA is shown by the induction of reverse mutations in the 2 sensitive *S. typhimurium* strains. This is the first demonstration that the liver microsomal metabolite of aflatoxin $B_1$ is not only toxic but mutagenic. It is surprising that both strains of *S. typhimurium* are mutated since the two strains are said to be mutated by different classes of mutagen, TA 1530 by agents causing base pair substitutions and TA 1531 by frame shift mutagens. Probably the metabolite can both intercalate in the bacterial DNA to reverse the original frame shift mutation and alkylate bases (guanine?) to cause a base pair substitution. The intercalation may be facilitated by the large planar shape of the aflatoxin molecule, the reactive grouping at the vinyl ether end of the molecule generated by metabolism then attacking a nucleophilic centre in the DNA.

Although previous data have shown that the aflatoxin $B_1$ metabolite is a reactive, labile molecule it was not possible to identify it. None of the known metabolites of aflatoxin $B_1$ were toxic to the bacteria. On the basis of structure activity studies with a number of aflatoxin congeners it was proposed that the reactive metabolite may be activated at the 2, 3 double bond and might possibly be 2,3-epoxyaflatoxin $B_1$. The tests using some other bisfuranoid compounds confirm that the vinyl ether grouping is essential for activity. The only exception to this is aflatoxin $B_2$. Although one cannot rule out the possibility that this was contaminated with trace amounts of aflatoxin $B_1$ (thin layer chromatography in $CHCl_3$/methanol (97 : 3) showed aflatoxin $B_1$ to have an $R_f$ value of 0·90 whereas aflatoxin $B_2$ had an $R_f$ of 0·77 with no visible contamination with aflatoxin $B_1$), it could be that there are dehydrogenases in the liver able to convert aflatoxin $B_2$ to aflatoxin $B_1$, as suggested by other workers (Wogan, Edwards and Newberne, 1971).

The metabolite appears not to be attacked by the type of nucleophiles which have shown reactivity towards the epoxides of polycyclic hydrocarbons. The only nucleophile which had any inhibitory action in the microsomal activation assay was 2,3-dimercaptopropanol. The inhibitory action of 2,3-dimercaptopropanol may be because this compound is readily soluble in the microsomal membranes and is able to attack the toxic metabolite almost immediately it is formed. The effect of dimercaptopropanol does not appear to be due to an inhibition of aflatoxin metabolism as experiments using microsomes, dimercaptopropanol and $^{14}C$ aflatoxin $B_1$ *in vitro* show no alteration in the amount of aflatoxin $B_1$ metabolized (Garner, unpublished). One can conclude, therefore, that the metabolite is reactive and that its formation is dependent on the vinyl ether group. Recent work has provided strong chemical evidence for the formation of 2,3-epoxy aflatoxin $B_1$ during microsomal metabolism; this may be the lethal and mutagenic metabolite (Garner, 1973*b*). On the basis of their studies other workers have also suggested that aflatoxin $B_1$ may be converted to an active metabolite (Goodall and Butler, 1969; Edwards and Wogan, 1970; Patterson and Roberts, 1970).

There is now good evidence that a correlation exists between compounds that induce mutations in bacteria and those which are carcinogenic (Miller and Miller, 1971). It would be surprising if the aflatoxin $B_1$ metabolite(s) which induces mutations in bacteria is in no way related to that which initiates cancer in animals. Studies with aflatoxin and related compounds clearly show the potential of these short-term tests using micro-organisms to detect activated metabolites. Of the compounds mentioned in this report, a number have not been tested for carcinogenicity (parasiticol, *O*-methyl sterigmatocystin, 5-methoxy sterigmatocystin and the versicolorins) although they may be ingested by man. It is therefore essential to look at these compounds in much more detail to evaluate any potential hazard to man.

This work was supported by a grant from the Yorkshire Council of the Cancer Research Campaign. We would like to thank Dr S. Baumberg for helpful discussions.

### REFERENCES

AMES, B. N. (1971) The Detection of Chemical Metagens with Enteric Bacteria. In *Chemical Mutagens. Principles and Methods for Their Detection*. Ed. A. Hollaender. New York: Plenum Press. Vol. 1, p. 267.

AMES, B. N., SIMS, P. & GROVER, P. L. (1971) Epoxides of Carcinogenic Polycyclic Hydrocarbons are Frameshift Mutagens. *Science, N.Y.*, **176**, 47.

AMES, B. N., GURNEY, E. G., MILLER, J. A. & BARTSCH, H. (1972) Carcinogens as Frameshift Mutagens: Metabolites and Derivatives of 2-Acetylaminofluorene and Other Aromatic Amine Carcinogens. *Proc. natn. Acad. Sci. U.S.A.*, **69**, 3128.

AMES, R. N., LEE, F. D. & DURSTON, W. E. (1973) An Improved Bacterial Test System for the Detection and Classification of Mutagens and Carcinogens. *Proc. natn. Acad. Sci. U.S.A.*, **70**, 782.

BRUCE, H. M. & PARKES, A. S. (1956) Letter. *J. anim. techns Ass.*, **7**, 54.

EDWARDS, G. S. & WOGAN, G. N. (1970) Aflatoxin Inhibition of Template Activity of Rat Liver Chromatin. *Biochim. biophys. Acta*, **224**, 597.

GABRIDGE, M. G. & LEGATOR, M. S. (1969) A Host-mediated Microbial Assay for the Detection of Mutagenic Compounds. *Proc. Soc. exp. Biol. Med.*, **130**, 831.

GARNER, R. C. (1973*a*) Microsome-dependent Binding of Aflatoxin $B_1$ to DNA, RNA, Polyribonucleotides and Protein *in vitro*. *Chem. biol. Interactions*, **6**, 125.

GARNER, R. C. (1973*b*) Chemical Evidence for the Formation of a Reactive Aflatoxin $B_1$ Metabolite, by Hamster Liver Microsomes. FEBS Letters, in press.

GARNER, R. C., MILLER, E. C., MILLER, J. A., GARNER, J. V. & HANSON, R. S. (1971) Formation of a Factor Lethal for *S. typhimurium* TA 1530 and TA 1531 on Incubation of Aflatoxin $B_1$ with Rat Liver Microsomes. *Biochem. biophys. Res. Commun.*, **45**, 774.

GARNER, R. C., MILLER, E. C. & MILLER, J. A. (1972) Liver Microsomal Metabolism of Aflatoxin $B_1$ to a Reactive Derivative Toxic to *Salmonella typhimurium* TA 1530. *Cancer Res.*, **32**, 2058.

GOLDBLATT, L. A. (1969) *Aflatoxin*. New York and London: Academic Press.

GOODALL, C. M. & BUTLER, W. H. (1969) Aflatoxin Carcinogenesis: Inhibition of Liver Cancer Induction in Hypophysectomised Rats. *Int. J. Cancer*, **4**, 422.

GROVER, P. L., HEWER, A. & SIMS, P. (1971) Epoxides as Microsomal Metabolites of Polycyclic Hydrocarbons. FEBS Letters, **18**, 76.

HOWARD-FLANDERS, P. (1968) DNA repair. *A. Rev. Biochem.*, **37**, 175.

IARC Monographs on the Evaluation of Carcinogenic Risk of Chemicals to Man, vol. 1. IARC, Lyon, 1972.

LAMB, M. J. & LILLY, L. J. (1971) Induction of Recessive Lethals in *Drosophila melanogaster* by Aflatoxin $B_1$. *Mutation Res.*, **11**, 430.

MAGEE, P. N. & SWANN, P. F. (1969) Nitroso Compounds. *Br. med. Bull.*, **25**, 240.

MAHER, V. M. & SUMMERS, W. C. (1970) Mutagenic Action of Aflatoxin $B_1$ on Transforming DNA and Inhibition of DNA Template Activity *in vitro*. *Nature, Lond.*, **225**, 68.

MALLING, H. V. (1971) Dimethylnitrosamine: Formation of Mutagenic Compounds by Interaction with Liver Microsomes. *Mutation Res.*, **13**, 425.

MARSHALL, W. J. & MCLEAN, A. E. M. (1969). The Effect of Oral Phenobarbitone on Hepatic Microsomal Cytochrome P-450 and Demethylation Activity in Rats Fed Normal and Low Protein Diets. *Biochem. Pharmac.*, **18**, 153.

MILLER, E. C. & MILLER, J. A. (1971) The Mutagenicity of Chemical Carcinogens: Correlations, Problems and Interpretations. In *Chemical Mutagens, Principles and Methods for their Detection*. Ed. A. Hollaender. New York and London: Plenum Press. Vol. I, p. 83.

MILLER, J. A. (1970) Carcinogenesis by Chemicals: An Overview. G. H. A. Clowes Memorial Lecture. *Cancer Res.*, **30**, 559.

ONG, T. & DE SERRES, F. J. (1972) Mutagenicity of Chemical Carcinogens in *Neurospora crassa*. *Cancer Res.*, **32**, 1890.

PATTERSON, D. S. P. & ROBERTS, B. A. (1970) The Formation of Aflatoxins $B_{2a}$ and $G_{2a}$ and Their Degradation Products during the *in vitro* Detoxification of Aflatoxin by Livers of Certain Avian and Mammalian Species. *Fd & cosmet. Toxicol.*, **8**, 527.

SHANK, R. C., WOGAN, G. N. & GIBSON, J. B. (1972) Dietary Aflatoxins and Human Liver Cancer. 1. Toxigenic Moulds in Foodstuffs of Tropical South-East Asia. *Fd & cosmet. Toxicol.*, **10**, 51.

SIMS, P. (1970) Qualitative and Quantitative Studies on the Metabolism of a Series of Aromatic Hydrocarbons by Rat Liver Preparations. *Biochem. Pharmac.*, **19**, 795.

TOYOSHIMA, K., HIASA, Y., ITO, N. & TSUBURA, Y. (1970) *In vitro* Malignant Transformation of Cells Derived from Rat Liver by Means of Aflatoxin $B_1$. *Gann*, **61**, 557.

TYRELL, R. M., MOSS, S. H. & DAVIES, D. J. G. (1972) The Variation in UV Sensitivity of Four K12 Strains of *Escherichia coli* as a Function of Their Stage of Growth. *Mutation Res.*, **16**, 1.

WOGAN, G. N. & NEWBERNE, P. M. (1967) Dose-response Characteristics of Aflatoxin $B_1$ Carcinogenesis in the Rat. *Cancer Res.*, **27**, 2370.

WOGAN, G. N., EDWARDS, G. S. & NEWBERNE, P. M. (1971) Structure–Activity Relationships in Toxicity and Carcinogenicity of Aflatoxins and Analogs. *Cancer Res.*, **31**, 1936.